# From species descriptions to diversity patterns: the validation of taxonomic data as a keystone for ant diversity studies reproducibility and accuracy

**DOI:** 10.1098/rsos.221170

**Published:** 2023-02-08

**Authors:** Rodrigo M. Feitosa, Thiago S. R. Silva, Gabriela P. Camacho, Mônica A. Ulysséa, Natalia Ladino, Aline M. Oliveira, Emília Z. de Albuquerque, Carla R. Ribas, Fernando A. Schmidt, Maria Santina de C. Morini, Rogério R. da Silva, Wesley Dáttilo, Antônio C. M. de Queiroz, Fabrício B. Baccaro, Jean C. Santos, Karine S. Carvalho, Tathiana G. Sobrinho, Yves P. Quinet, Aline B. Moraes, André B. Vargas, Helena Maura Torezan-Silingardi, Jorge Luiz P. Souza, Tatianne Marques, Thiago Izzo, Denise Lange, Iracenir A. dos Santos, Kleber Del-Claro, Larissa Nahas, Lucas Paolucci, Stela A. Soares, Ana Y. Harada, Ananza M. Rabello, Cinthia B. da Costa-Milanez, Eduardo Diehl-Fleig, Renata B. F. Campos, Ricardo Solar, Tiago Frizzo, Wesley DaRocha, Anselmo Nogueira

**Affiliations:** ^1^ Laboratório de Sistemática e Biologia de Formigas, Departamento de Zoologia, Universidade Federal do Paraná, Curitiba, Paraná, Brazil; ^2^ The Insect Biodiversity and Biogeography Laboratory, School of Biological Sciences, The University of Hong Kong, Hong Kong SAR, People's Republic of China; ^3^ Center for Integrative Biodiversity Discovery, Museum für Naturkunde, Berlin, Germany; ^4^ Laboratório de Hymenoptera, Museu de Zoologia da Universidade de São Paulo, São Paulo, Brazil; ^5^ AntLab, National Museum of Natural History, Smithsonian Institution, Washington, DC, USA; ^6^ Rabeling Lab, School of Life Sciences, Arizona State University, Tempe, AZ, USA; ^7^ Laboratório de Ecologia de Formigas, Departamento de Ecologia e Conservação, Universidade Federal de Lavras, Lavras, Minas Gerais, Brazil; ^8^ Lancaster Environment Centre, Lancaster University, Lancaster, UK; ^9^ Laboratório de Ecologia de Formigas, Centro de Ciências Biológicas e da Natureza, Universidade Federal do Acre, Rio Branco, Acre, Brazil; ^10^ Laboratório de Mirmecologia do Alto Tietê, Núcleo de Ciências Ambientais, Universidade de Mogi das Cruzes, Mogi das Cruzes, São Paulo, Brazil; ^11^ Coordenação de Ciências da Terra e Ecologia, Museu Paraense Emílio Goeldi, Belém, Pará, Brazil; ^12^ Red de Ecoetología, Instituto de Ecología AC, Xalapa, Veracruz, Mexico; ^13^ Departamento de Biologia, Universidade Federal do Amazonas, Manaus, Amazonas, Brazil; ^14^ Laboratório de Ecologia & Biodiversidade, Departamento de Ecologia, Universidade Federal de Sergipe, São Cristóvão, Sergipe, Brazil; ^15^ Laboratório de Ecologia, Departamento de Ciências Naturais, Universidade Estadual do Sudoeste da Bahia, Campus Vitória da Conquista, Vitória da Conquista, Bahia, Brazil; ^16^ Laboratório de Sistemática e Ecologia de Insetos, Departamento de Ciências Agrárias e Biológicas, Universidade Federal do Espírito Santo, Campus São Mateus, São Mateus, Espírito Santos, Brazil; ^17^ Laboratório de Entomologia, Departamento de Biologia, Universidade Estadual do Ceará, Fortaleza, Ceará, Brazil; ^18^ Prefeitura Municipal de Novo Hamburgo, Novo Hamburgo, Rio Grande do Sul, Brazil; ^19^ Centro Universitário de Volta Redonda – UniFOA, Volta Redonda, Rio de Janeiro, Brazil; ^20^ Laboratório de Ecologia Comportamental e de Interações, Instituto de Biologia. Universidade Federal de Uberlândia, Uberlândia, Minas Gerais, Brazil; ^21^ Instituto Nacional da Mata Atlântica – INMA, Santa Teresa, Espírito Santo, Brazil; ^22^ Laboratório de Ecologia Aplicada e Citogenética, Instituto Federal do Norte de Minas Gerais – IFNMG, Campus Salinas, Salinas, Minas Gerais, Brazil; ^23^ Laboratório de Ecologia de Comunidades, Departamento de Botânica e Ecologia, Universidade Federal do Mato Grosso, Cuiabá, Mato Grosso, Brazil; ^24^ Universidade Tecnológica Federal do Paraná, Campus Santa Helena, Santa Helena, Paraná, Brazil; ^25^ Centro de Formação Interdisciplinar, Universidade Federal do Oeste do Pará, Santarém, Pará, Brazil; ^26^ Departamento de Biologia Geral, Universidade Federal de Viçosa, Viçosa, Minas Gerais, Brazil; ^27^ Secretaria Estadual de Educação de Mato Grosso do Sul, Campo Grande, Mato Grosso do Sul, Brazil; ^28^ Coordenação em Zoologia, Museu Paraense Emilio Goeldi, Belém, Pará, Brazil; ^29^ Instituto de Estudos do Xingu, Universidade Federal do Sul e Sudeste do Pará, São Félix do Xingu, Pará, Brazil; ^30^ Departamento de Biologia, Instituto de Ciências Exatas e Biológicas, Universidade Federal de Ouro Preto, Ouro Preto, Minas Gerais, Brazil; ^31^ In Memoriam, São Leopoldo, Rio Grande do Sul, Brazil; ^32^ Laboratório de Ecologia, Ambiente e Território, PPG Gestão Integrada do Território, Universidade Vale do Rio Doce, Governador Valadares, Minas Gerais, Brazil; ^33^ Centro de Síntese Ecológica e Conservação, Departamento de Genética. Ecologia e Evolução, Universidade Federal de Minas Gerais, Belo Horizonte, Minas Gerais, Brazil; ^34^ Laboratório de Ecologia de Insetos, Departamento de Biologia Geral, Universidade Federal de Minas Gerais, Belo Horizonte, Minas Gerais, Brazil; ^35^ Departamento de Ecologia, Instituto de Ciências Biológicas, Universidade de Brasília, Campus Darcy Ribeiro, Asa Norte. Brasília, Distrito Federal, Brazil; ^36^ Laboratório de Mirmecologia, Centro de Pesquisa do Cacau, Ilhéus, Bahia, Brazil; ^37^ Laboratório de Interações Planta-Animal (LIPA) – Centro de Ciências Naturais e Humanas, Universidade Federal do ABC, São Bernardo do Campo, São Paulo, Brazil

**Keywords:** biological collections, community ecology, scientific reproducibility, scientometrics, vouchers, myrmecology

## Abstract

Research findings in natural sciences need to be comparable and reproducible to effectively improve our understanding of ecological and behavioural patterns. In this sense, knowledge frontiers in biodiversity studies are directly tied to taxonomic research, especially in species-rich tropical regions. Here we analysed the taxonomic information available in 470 studies on Brazilian ant diversity published in the last 50 years. We aimed to quantify the proportion of studies that provide enough data to validate taxonomic identification, explore the frequency of studies that properly acknowledge their taxonomic background, and investigate the primary resources for ant identification in Brazil. We found that most studies on Brazilian ant diversity (73.6%) explicitly stated the methods used to identify their specimens. However, the proportion of papers that provide complete data for the repository institutions and vouchered specimens is vanishingly small (5.8%). Additionally, only 40.0% of the studies consistently presented taxon authorities and years of description, rarely referencing taxonomic publications correctly. In turn, the number of specialists and institutions consulted for ant identification in Brazil has increased in the last years, along with the number of studies that explicitly provide their taxonomic procedures for ant identification. Our findings highlight a shift between generations regarding the recognition of taxonomy as fundamental science, deepening our understanding of biodiversity.

## 1. Introduction

Earth's biodiversity faces an unprecedented threat represented mainly by the mass extinction of natural populations and resources, climate change and the pollination crisis [1–4]. This threat is especially true for natural habitats under high anthropic pressure, primarily concentrated in tropical countries [5]. These areas hold most species on the planet, many of which are likely to be extinct even before their formal description [6,7]. On the other hand, the current existence of a formal description for part of this biodiversity does not guarantee that ecological surveys correctly apply their formal names. In this scenario, precise and comprehensive approaches are urgently needed to describe, measure and protect biodiversity. Effective biodiversity conservation depends on surveying, monitoring, analysing and reacting to changes in natural systems, most of which focus on community and population ecology [8]. Such efforts are highly dependent on species composition surveys on different spatial and temporal scales, a task broadly based on local diversity studies highly dependent on the taxonomic precision achieved. Within a scenario in which a large set of new data about ecological patterns and conservation status becomes available daily for different taxonomic groups, a crucial question emerges: how do these researchers obtain the exact scientific names for all organisms they are dealing with?

Misidentified taxa in observational and manipulative experiments may compromise biological interpretations and comparisons across studies. Primarily, the propagation of such errors can affect our estimations of the relative abundance, diversity, and even the distribution of native and non-native organisms [9,10]. The consequences of such taxonomic errors can be significantly diverse, including social, sanitary and economic effects, negatively impacting human well-being [11].

Regarding ecological studies and diversity inventories, incorrect or obsolete taxonomy can lead to a lack of congruence among existing surveys and the assembly of taxonomically unreliable datasets, as demonstrated by Jansen & Dengler [12] when exploring neglected sources of taxonomic bias in plant datasets. Ultimately, unreliable diversity datasets increase the risk of reaching erroneous ecological conclusions and making poorly informed conservation and management decisions [13]. In another example, a recent survey demonstrated that around 7.5% of the ecological studies dealing with Brazilian ant diversity published from 1970 to 2021 presented inconsistencies in their species lists regarding the validity of taxonomic names and geographically implausible taxa records. Some of these taxonomically spurious names have been propagated by subsequent studies, causing non-existent species to compose local lists of taxa or even support conclusions about ant diversity patterns in Brazil [14]. In this sense, precision regarding identification methods and taxon concepts is fundamental to avoid errors from species-specific findings to community-level hypotheses. Thus, the requirement of explicit information about identification should be the baseline for assessing a study's reproducibility [15].

Accurate, available and reproducible taxonomic information (in the form of data and metadata) is the hallmark of science communication, without which biological research could become invalid [9]. Nonetheless, not every researcher can be a specialist on the subject taxa, relying mainly on using previously published identification keys or collaborating with taxonomists. Even following this path, several studies failed to provide basic taxonomic details as the taxonomic methods (source of taxonomic literature used for identification, reference collections and specialists consulted), reproducibility of the taxonomic decisions (specimen vouchering and explicit mention of depositary collections) and recognition of taxonomic agents (proper citation of taxonomic works, taxa authorities and explicit reference to specialists' names and institutions) [15–18]. The absence of such information prevents the precise validation of taxonomic identification and the confirmation or refutation of any ecological study's results. To explore how taxonomic research output is applied and cited by scientists, we analysed a comprehensive dataset of ant diversity studies produced by the world's second-largest myrmecological community in number of researchers, Brazil [19,20]. With more than 8.5 million km^2^, Brazil occupies a large area along the eastern coast of South America and includes much of the continent's interior. Within its vast geographical range and six official biomes, the country encompasses remarkable heterogeneity in topography, climate, soil, vegetation and hydrography [21]. This notable variation in tropical and subtropical habitats is also reflected in Brazil's biodiversity, which is considered the highest in the world [22,23]. The same diversity pattern applies to the Brazilian ant fauna, which represents about one third of the genera (117 out of 345) and one tenth of the species known to occur on the planet (1500 out of 14 000) [24,25].

The considerable accumulated knowledge about ants in Brazil is largely due to the major role these social insects play in terrestrial ecosystems [26] and to the historical taxonomic and ecological research effort initiated in the nineteenth century [19,27,28]. This effort is currently represented by approximately 70 research groups and more than 500 scientists spread across all the country's regions, whose main research topic is community ecology [20,29,30]. Therefore, it is expected that much of the knowledge produced by these researchers depends largely on a precise taxonomic background to enable scientific communication. With that in mind, we aim to provide baseline data on a comprehensive range of taxonomic tools used by myrmecologists by surveying all studies published in the last 50 years investigating any aspect of ant diversity in Brazil. We specifically ask how identification procedures, specimen vouchering and recognition of the taxonomic routine evolved within the Brazilian myrmecological community in the last five decades. Based on the information obtained in this study, scientific practices and temporal trends regarding the application of taxonomic data can guide the academic and editorial policies of funding agencies and scientific journals. We also highlight that our conclusions can increase the commitment of researchers worldwide to the replicability of taxonomic data in non-taxonomic studies, especially those dealing with biodiversity conservation initiatives implementing species monitoring. Finally, we hope to encourage the appropriate recognition of taxonomy as a fundamental science to biology, thus reducing the distance between taxonomists and other researchers regarding academic metrics.

## 2. Material and methods

### 2.1. Literature search

We considered studies dealing specifically with ant diversity (Hymenoptera: Formicidae) across the Brazilian terrestrial biomes. The keywords employed in the literature search included ‘ant + Brazil’ or ‘*formiga* + *Brasil*’ (in Portuguese). Secondarily, we performed searches using these same keywords followed by the name of each Brazilian biome (Amazon Forest, Atlantic Forest, Caatinga, Cerrado, Pampa and Pantanal, following [21]). After searches, only studies dealing with the Brazilian ant diversity were included in our dataset. We considered ‘ant diversity’ in a broad sense, including myrmecological surveys; checklists of ants in ecoregions, conservation unities and geopolitical provinces (states and municipalities); ecological interactions; behaviour biology; ecological and evolutionary aspects of ant assemblages (population, community, and conservation ecology); and studies on ant sampling techniques. Including the Brazilian biomes in the search terms was planned to meet the criteria of a previous comprehensive effort to describe the profile of ant diversity studies in Brazil (see [30]).

We performed standardized searches in three scientific databases, namely Web of Science (http://www.webofknowledge.com), SciELO (https://scielo.org/) and Scopus (https://www.scopus.com). The time range was from 1945 until May 2021. At the end of each online database search, a round of verification for the redundancy of studies was performed. Uncorrected proofs, online first versions of accepted manuscripts, and preprints were replaced by the final versions of articles whenever possible. Books, book chapters, event presentations, technical reports, taxonomic articles, studies on a single focal taxon and graduate dissertations were excluded from the final database.

### 2.2. Information survey

For each study on our dataset, we extracted 19 descriptors (electronic supplementary material, table S1). Three are reference components (authors, year and title), and 16 refer to taxonomic variables. In addition to searching through the main sections of the target papers, we also verified the acknowledgements where taxonomic experts are frequently named and the supplementary material where species or genera lists are commonly provided.

To describe how taxonomic procedures for ant identification are applied for ant diversity studies in Brazil, the 16 taxonomic descriptors defined here were sorted into four primary categories based mainly on the scheme proposed by [31]: (i) basic taxonomic information; (ii) taxonomic methods; (iii) reproducibility of taxonomic decisions; and (iv) taxonomy recognition.

The basic taxonomic information (category ‘i’) encompasses the presence and location of the taxa list in the manuscript (i.e. if available in the body text or as supplementary material). We considered a list as ‘present’ when the species' names were included in a taxonomic table or were simply mentioned sequentially in the text. This category also assessed if there was an explicit mention of the taxonomic classification adopted, represented by the citation of taxonomic synopses or catalogues in use by the time of the article's publication. In category ‘i’ we also considered the indication of taxa authorities (i.e. authors responsible for the original description of any given taxon), including their names and/or year of original descriptions. The indication of taxa authorities and year of description as a variable is motivated by the fact that the lack of citation of a species author and year of publication, or the referencing of taxonomic work in the main text or supplementary material, gradually increases the academic metric's distance (e.g. impact factors) between taxonomists and other researchers. This distance may explain the lack of interest in taxonomy by young scientists and the decline of specialists available for taxa identification, directly affecting taxonomic resolution in diversity studies [32].

For the taxonomic methods (category ‘ii’), we considered all the available tools and procedures for ant identification and/or validation of the taxa listed in the studies. These procedures include the use of taxonomic literature cited in the Material and Methods section (hereafter M&M) of studies (i.e. publications providing identification keys and/or taxa diagnoses and descriptions); reference ant collections used for taxa comparison mentioned in the M&M section (i.e. well-established collections of broad access listed in the literature and holding representative specimens of the local ant fauna); and specialists consulted for taxonomic identification mentioned either in the M&M or Acknowledgements. Specialists included researchers whose main investigation line is ant systematics (according to their master's or PhD projects) or experienced ant identifiers working on different knowledge areas (according to their names in the literature).

To increase the accuracy of taxonomic work citations, in two cases, we considered that different citations refer to the same work, even though the dates and titles assigned for these publications are not the same among different studies. These discrepancies occurred with Bolton's Ant Catalogue [24], originally published in 2006 and updated in its online version since 2013, and with the identification keys for Neotropical ants by Palacio & Fernández (*Claves para las subfamilias y géneros*), published as a chapter of the book *Introducción a las Hormigas de la Región Neotropical*, whose citation in literature is as much referenced to the book as to the chapter [33].

For category ‘iii’, named ‘reproducibility of taxonomic decisions', we considered information on the vouchering of physical specimens, either stored in ethanol or dry-mounted, including specimen identifier codes (specimen accession numbers). We also searched studies for the explicit mention of depositary collections defined for the reference collections in category ‘ii’.

Finally, for the ‘taxonomy recognition’ in category ‘iv’, we checked for the explicit mention of specialists’ names and institutions consulted as defined in category 'ii'; the proper citation in the references of the taxonomic works that underpinned the studies' conclusions, including original descriptions of taxa (represented by their taxonomic authorities in the text); and the presence of taxonomists among the authors, except in the cases where taxonomists were the first authors of diversity studies so that the authorship represents a true collaboration between research groups. According to their primary research interest, taxonomists here were researchers whose main investigation line is ant systematics, based on their master's or PhD projects.

### 2.3. Statistical analyses and data visualization

We used a time series analysis based on the arithmetic mean over past observations to explore temporal patterns of citation and mention of taxonomic works and authorities yearly. We initially calculated the frequency of mentions (%) for each taxonomic descriptor by dividing the absolute number of mentions by the total number of ant diversity studies published per year multiplied by 100. We performed temporal analyses only from 1994 onwards, given that only after that year, we had four or more studies published annually to calculate the frequency of mentions adequately. Therefore, we obtained the yearly percentage of mentions of each taxonomic descriptor that was not confounded with the temporal scaling pattern of the number of published ant diversity studies. To access trends in mentions of taxonomic publications and consulted collections, we considered them mentionable from the year of publication and establishment, respectively. All analyses and plotting were performed using the software R v. 4.2.1 [34]. In all cases, we modelled the percentage of mentions of each taxonomic variable using the function *SMA* in the package ‘TTR’ (Technical Trading Rules, [35]). We plotted the graphs using the ‘ggplot2’ package [36]. We used five past observations for each time series analysis to calculate the averages over time, applying a simple moving average for smoothing the temporal patterns. As the time series modelling revealed complex nonlinear patterns of variation in the frequency of mentions, we were prevented from applying linear statistical analyses (e.g. simple regression) to complement the time series analyses. So, we compared the frequencies of mentions at two distinct periods using a binomial proportion test with the R function *prop.test* [37]. We compared the two extreme periods along the time series to complement the time series modelling. The first period was from 1994 to 1998, and the second was from 2017 to 2021. We considered that the frequency of mentions was different between the two periods if the *p*-value was less than 0.05 significance level. It is important to emphasize that our analyses cannot identify seasonal components over time (i.e. the intra-year fluctuations), only inter-year temporal patterns.

To describe the pattern of collaboration network among authors in the ant diversity studies, myrmecological collections and specialists, the data collected in our study were imported into Sci2 Tool (Science of Science Tool v. 1.3, https://sci2.cns.iu.edu). Node values (i.e. central coordinates of first authors' and specialists’ municipalities and collections' locations) and edge values (the number of collaborations between first authors and collections and specialists) were obtained using the ‘Extract Directed Network’ function and used to determine the institutional directionality graphically through in-degree values of all nodes. We imported node and edge values to the visualization tool Gephi (Gephi v. 0.9.2, http://gephi.org.) and built a network of global institutional collaboration using the ‘Geo Layout’ plugin. The obtained graph was exported through the open-source image editor Inkscape 1.1.1. as a vector graphic and plotted over the same scale world map.

## 3. Results

**Figure 1.**
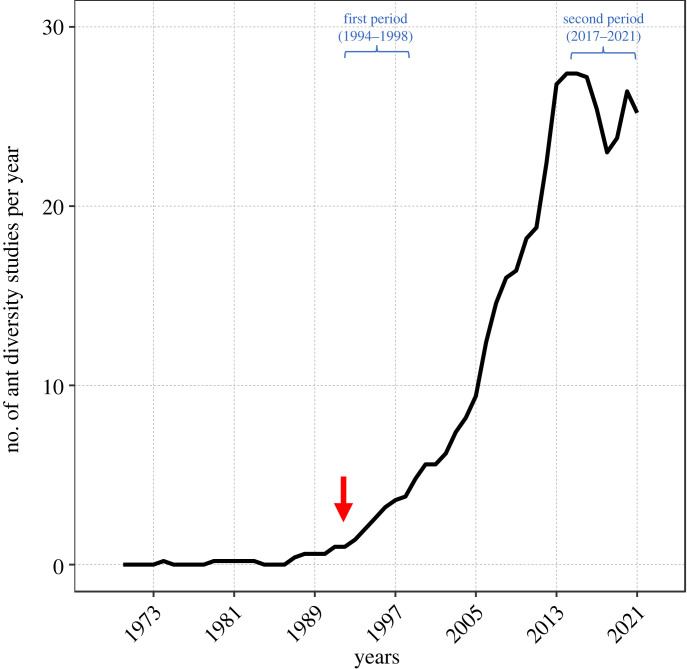
Number of ant diversity studies in Brazil published in the last 50 years recovered in our systematic literature review. The trend line is based on the arithmetic mean of the number of biodiversity studies over the past five years (n observations) using the function SMA of the R package TTR (Technical Trading Rules, [35]). The red arrow indicates 1994, when we had more than four ant diversity studies published yearly. From that year onwards, we investigated the temporal patterns of variation of the taxonomic data presented in figure 2. The periods highlighted in blue are the two moments in which we compared the frequency of mentions in the binomial proportion tests.

**Figure 2.**
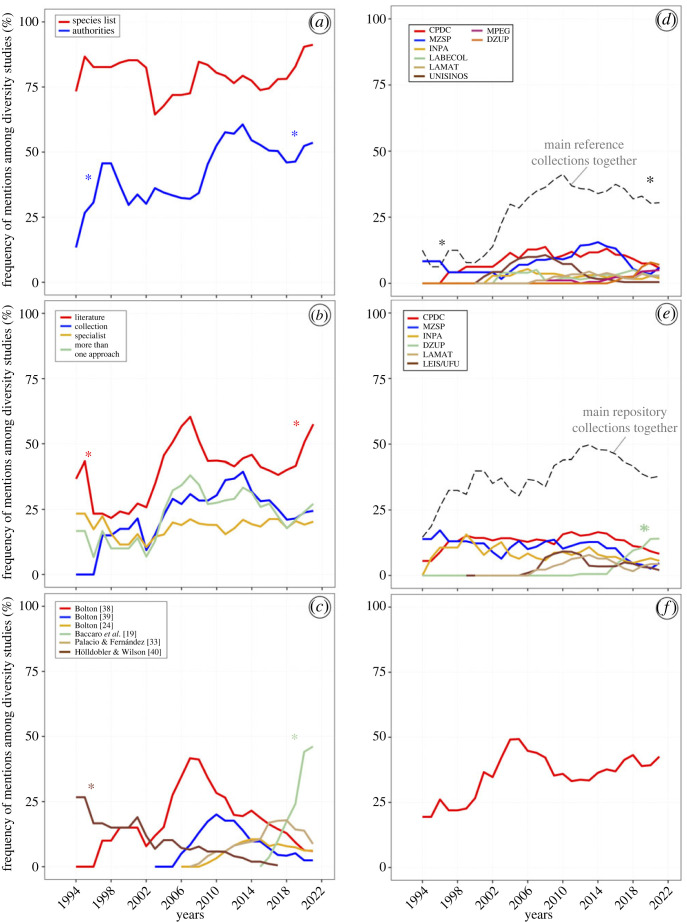
Frequency of mentions of taxonomic data over the years obtained from ant diversity studies in Brazil. The trend lines are based on time series modelling and describe the frequency of mentions based on the arithmetic mean of the series over the past five observations using the function SMA of the R package TTR (Technical Trading Rules, [35]). From top to bottom: (*a*) frequency of studies providing ant species list and explicitly mentioning taxa authorities; (*b*) frequency of studies that provided the methods for taxonomic identification; (*c*) frequency of mentions for the six most cited taxonomic publications; (*d*) frequency of mentions for the six most cited reference collections; (*e*) frequency of mentions for the six main repository collections that received ant vouchers; (*f*) frequency of mentions of ant specialists consulted for taxonomic identification. CPDC = *Centro de Pesquisas do Cacau*, Ilhéus, BA; MZSP = *Museu de Zoologia da Universidade de São Paulo*, São Paulo, SP; INPA = *Instituto Nacional de Pesquisas da Amazônia*, Manaus, AM; DZUP = *Coleção Entomológica Padre Jesus Santiago Moure da Universidade Federal do Paraná*, Curitiba, PR; LABECOL = *Laboratório de Ecologia de Comunidades da Universidade Federal de Viçosa*, Viçosa, MG; LAMAT = *Laboratório de Mirmecologia do Alto Tietê – Universidade de Mogi das Cruzes*, Mogi das Cruzes, SP; LEIS/UFU = *Laboratório de Ecologia de Insetos Sociais da Universidade Federal de Uberlândia*, Uberlândia, MG; MPEG = *Museu Paraense Emílio Goeldi*, Belém, PA; UNISINOS = *Laboratório de Genética de Insetos Sociais da Universidade do Vale do Rio dos Sinos*, São Leopoldo, RS. The asterisks indicated differences detected by the binomial proportion test comparing the frequency of mentions in the two extreme periods of the time series modeling. In cases where only one asterisk was associated with the trend line, the main proportion was compared with a null proportion (zero). We added asterisks only in tests where the *p*-value was less than 0.05 of the significance level. The values of statistics are available in the main text.

We compiled 491 studies on ant diversity from online databases. After applying our selection criteria and the validation rounds, we remained with 470 studies, from which we extracted the data analysed here. Regarding the time range of our final database, the first study was published in 1970 and the last in May 2021 (electronic supplementary material, table S1). From the 1990s onwards, we had an exponential increase in publications of ant diversity studies in Brazil (figure 1). Thus, we only explored temporal patterns by frequency (%) after this year (figure 2). To access the non-taxonomic information from the studies published from 1970 to 2020, including those not considered here, see the supporting information in [30].

### 3.1. Basic taxonomic information

A total of 390 studies (83.0%) presented a complete or partial list of the species sampled located in the main text (83.8%) or the supplementary material (16.2%). The frequency of studies providing the list of ant species since 1994 was around 80% on average, without showing a clear trend over time (figure 2*a*, red line, proportion binomial test: *χ*^2^ = 0.9, *p* = 0.167). From all the studies accessed, only 19.1% explicitly mentioned the taxonomic classification adopted. In many cases, taxonomic catalogues or synopses were also inadvertently cited as the main reference for taxonomic identification. Regarding the species authority information for those studies that mention species names, 59.7% of the studies explicitly provided the taxonomic authorities for the species listed. Of those, the description date (year) was given in 81.1% of studies, representing only 40.2% of our complete database. The frequency of studies explicitly providing taxonomic authorities increased noticeably over time, rising from 15% in 1994 to around 50% more recently (figure 2*a*, blue line, proportion binomial test: *χ*^2^ = 4.6, *p* = 0.016).

### 3.2. Taxonomic methods

A total of 73.6% of studies explicitly presented the taxonomic procedures adopted for achieving taxonomic identification, including taxonomic literature, specialists’ support, reference collections or combinations of these methods. From these, 68.2% of studies relied on the taxonomic literature for ant identification, with 1 to 15 taxonomic references consulted per study. The frequency of studies relying on the taxonomic literature for ant identification increased very slightly over time, rising from 30–40% around 1994 to 50% more recently (figure 2*b*, red line, proportion binomial test: *χ*^2^ = 9.7, *p* < 0.001), although with many variations over time. In total, 105 taxonomic references were mentioned in the ant diversity studies accessed here (electronic supplementary material, table S2).

The prominent taxonomic references consulted were: (1) ‘Bolton, 1994. *Identification guide to the ant genera of the world*’ [38] (84 citations); (2) ‘Baccaro *et al.,* *Guia para os gêneros de formigas do Brasil*’ [19] (61 citations); (3) ‘Palacio & Fernández, 2003. *Claves para las subfamilias y géneros*’ [33] (38 citations); (4) ‘Bolton, 2003, *Synopsis and classification of Formicidae*’ [39] (35 citations); and (5) ‘Bolton, 2006–2022. *An online catalog of the ants of the world*’ [24] (25 citations). However, the author's references were not used with the same frequency over time (figure 2*c*). For example, Bolton 1994 and 2003 had a peak of use around 2008 and 2011, respectively, but after they have been less used over time (figure 2*c*, red and blue lines; proportion binomial test: *χ*^2^ = 2.2, *p* = 0.93 and *χ*^2^ = 0.1, *p* = 0.50, respectively). In addition, the work of Hölldobler & Wilson 1990 [40], which was widely used in 1994 (25% of studies), is no longer used today (figure 2*c*, brown line, proportion binomial test: *χ*^2^ = 10.3, *p* < 0.001). Finally, after the publication of Baccaro *et al*. [19], there was an exponential increase in the frequency of use of this reference, reaching almost 50% of studies more recently (figure 2*c*, green line, proportion binomial test: *χ*^2^ = 15.6, *p* < 0.001).

A total of 39.0% of studies indicated that the taxa were identified by comparison with reference ant collections, totalling 210 ant collection mentions (electronic supplementary material, table S3). The frequency of studies that identified ant species by comparison with ant collections slightly increased, from 0% in 1994 to around 25% more recently, although with many variations over time (figure 2*b*, blue line). This variation prevented us from detecting differences in the binomial proportion test (*χ*^2^ = 0.25, *p* = 0.308). Two of the 35 collections stand out for the higher frequency of mentions than other institutions. The myrmecological collections of the *Centro de Pesquisas do Cacau – Comissão Executiva do Plano da Lavoura Cacaueira* (CPDC) and the *Museu de Zoologia da Universidade de São Paulo* (MZSP) were consulted for ant identification/confirmation in Brazil in 43 and 42 studies, respectively. Together, both institutions represent 40.5% of the identifications by comparison in ant collections in Brazil, followed by *Coleção de Insetos do Instituto Nacional de Pesquisas da Amazônia* (INPA) (13 citations), *Laboratório de Ecologia de Comunidades da Universidade Federal de Viçosa* (LABECOL) (12 citations), *Coleção de Formigas do Laboratório de Mirmecologia do Alto Tietê – Universidade de Mogi das Cruzes* (LAMAT) (11 citations), *Coleção Entomológica do Museu Paraense Emílio Goeldi* (MPEG) (11 citations), *Coleção de Formicidae do Laboratório de Genética de Insetos Sociais da Universidade do Vale do Rio dos Sinos* (UNISINOS) (11 citations), and *Coleção Entomológica Padre Jesus Santiago Moure da Universidade Federal do Paraná* (DZUP) (10 citations) (figure 2*d*). However, there was no marked temporal pattern of citations of each of the six most important collections for comparing specimens for ant identification (figure 2*d*). Considering all six collections together, a pattern of increasing use of reference collections for ant identification becomes apparent over time, rising from around 12% in 1994 to 35% more recently, with a peak of 40% in 2010 (figure 2*d*, dashed black line, proportion binomial test: *χ*^2^ = 61.8, *p* < 0.001).

Finally, 40.0% of studies declared that the ant species sampled were identified with the aid of specialists (taxonomists or experienced identifiers), representing 381 mentions in the literature. The frequency of studies declaring that a specialist identified the ant species varied little since 1994, remaining relatively constant between 15 and 22% of studies (figure 2*b*, orange line, proportion binomial test: *χ*^2^ = 0.15, *p* = 0.349). A total of 82 researchers are listed as ant identifiers in studies published on Brazilian ant diversity (electronic supplementary material, table S4), and around 30.4% had the same two specialists as ant identifiers, namely Rodrigo M. Feitosa from DZUP (59 mentions) and Jacques H. C. Delabie from CPDC (57 mentions).

### 3.3. Reproducibility of the taxonomic decisions

Overall, 66.0% of studies clearly stated that the specimens collected were formally vouchered, with only 5.8% including the accession codes of specimens in the text or appendages. All the studies whose specimens were vouchered mentioned the depository collections. However, several studies failed to provide the collection name and location (i.e. the internal divisions and complete addresses of institutions).

In total, 66 depository collections were mentioned (electronic supplementary material, table S5), totalling 365 citations of collections in the literature. Thirty-four collections are considered institutional, whereas 31 are restricted to research laboratories, and one is considered private (Harold G. Fowler collection). There was an increase in the number of institutions available for voucher depositing in Brazil in the last two decades. However, six collections were the most important voucher depository institutions (figure 2*e*). Three collections stand out for having accumulated more than 30 citations: CPDC (58 citations), MZSP (43 citations) and INPA (37 citations). However, the frequency of use of these three institutions has remained constant, with a slight and gradual reduction of voucher depositing in these three collections in the last 10 years (figure 1*e*, red, blue, and orange lines). In these three cases, the binomial proportion test did not detect any differences in the frequency of mentions. Oppositely, since 2015 there has been an increase in voucher depositing in the DZUP collection (figure 2*e*, green line, proportion binomial test: *χ*^2^ = 2.7, *p* = 0.051). Considering the six most important repository collections together, there was a slight increase in the frequency of use of deposit collections for ant identification over time, although not detected in the binomial proportion test (figure 2*e*, dashed black line, *χ*^2^ = 0.1, *p* = 0.50).

### 3.4. Taxonomy recognition

Considering those studies that stated that the ants collected were identified with the aid of taxonomists, 98.4% mentioned the name of the specialist either in the M&M or Acknowledgements. Still, most studies (65.1%) did not provide the institutional affiliations of these specialists. The frequency of studies that cited a specialist for ant identification increased slightly over time, rising from 20–24% in 1994 to 40% more recently, although with many variations over time (figure 2*f*, red line). This variation prevented us from detecting differences in the binomial proportion test (*χ*^2^ = 1.8, *p* = 0.09).

All studies that relied on the taxonomic literature to identify ant taxa properly cited the works in their references. On the other hand, none of the 189 studies that provided the taxa authorities (i.e. name of the author of original description) included their taxonomic references.

**Figure 3.**
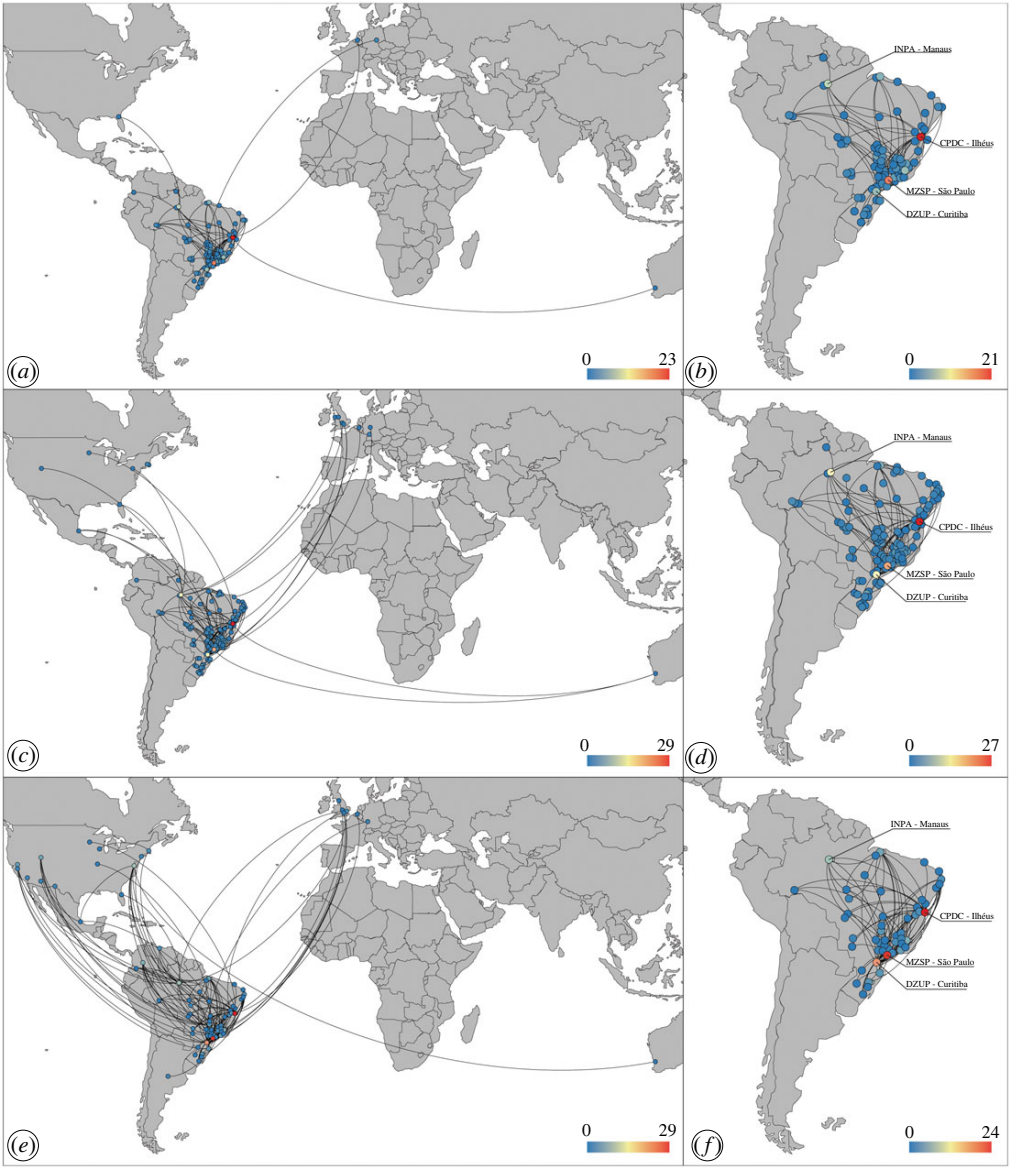
Collaboration maps in the myrmecology field based on ant diversity studies in Brazil, including international networks. The circles represent authors, identification specialists, and collections localities, while the lines represent collaborations. Collaboration directionality (i.e. the direction of collaboration from source locality to target locality) is represented by clockwise curved lines. Redder circles represent localities with high values of collaboration (defined by high in-degree values), while bluer circles represent localities with low values of collaboration (defined by low-in-degree values). (*a*), (*c*) and (*e*) collaborations between authors and reference collections on a global scale; (*b*), (*d*) and (*f*) collaborations between authors and reference collections in Brazil. The four Brazilian institutions with higher in-degree values are indicated in maps (*b*), (*d*) and (*f*). Abbreviations: CPDC, *Coleção de Formicidae do Centro de Pesquisas do Cacau*; DZUP, *Coleção Entomológica Padre Jesus Santiago Moure da Universidade Federal do Paraná*; INPA, *Coleção de Insetos do Instituto Nacional de Pesquisas da Amazônia*; MZSP, *Acervo de Formicidae do Museu de Zoologia da Universidade de São Paulo*.

According to the authors’ affiliation data and the location of identifier specialists and collections, Brazil's network on ant diversity involved 118 institutions from 11 countries worldwide over the last 50 years (figure 3*a,c*,*e*). A total of 28.7% of studies on ant diversity in Brazil had taxonomists among their authors. Finally, the highest levels of collaborative relationships are centred in seven Brazilian institutions, namely the DZUP, CPDC, MZSP, MPEG, INPA, LABECOL and the *Laboratório de Ecologia de Insetos Sociais da Universidade Federal de Uberlândia* (LEIS/UFU) (figure 3*b*,*d*,*f*).

## 4. Discussion

The value of any ecological dataset and, consequently, scientific products' credibility depends on the taxonomic accuracy of the names included therein [41]. In addition, the reproducibility of studies involving any aspect of conservation ecology, diversity and behavioural patterns requires that the methods used to identify organisms be explicitly stated and the material studied be available for further verification [9,31]. In this sense, most studies on ant diversity published in Brazil from 1970 to 2021 accomplished these criteria. In total, 73.6% of studies overtly stated the methods used for ant identification, while 66.0% detailed voucher specimen deposition (figure 2*b*,*e*), though rarely including the accession codes. Another positive trend observed is that more than 80.0% of the studies listed the species sampled. These proportions are considerably higher than those found by Packer *et al*. [31] based on evaluating all studies published in nine entomological journals in 2016. Those authors found that only 28.7% of studies declared the identification methods used, and no more than 23.6% vouchered the identified ant specimens. Similar results emerged from a comprehensive survey by Bortolus [10] that found 62.5% of the 80 studies published in ecological journals without any information about how the names of their study organisms were obtained. Our findings are even more relevant when we consider that the authors mentioned above found these patterns based on a temporally restricted dataset (one to two years) and on a limited number of journals (one to nine). Here, we have gathered data from half a century of information published in over 130 journals.

Despite the apparent taxonomic commitment of ant researchers in Brazil, the numbers obtained here also show that around 30.0% of the studies fail to meet the minimum reproducibility criteria (i.e. the taxonomic methods applied for achieving taxa identification). Also, only 40.2% correctly provided the taxa authorities and the species descriptions' year. Still, only 19.1% of studies mentioned the taxonomic classification adopted. Some researchers could argue that the year of publication would be enough to inform the readers about the classification system applied, given that the authors tend to adopt the most up-to-date valid classification. However, classifications are not always universal, and different proposals may be current at a given period for different taxonomic categories (i.e. tribes and genera). In addition, species identification is usually performed before publication, and the taxonomic classifications can eventually change, rendering the taxonomy of these studies obsolete.

The primary source for identifying ants collected in diversity studies in Brazil is the taxonomic literature, with around 50.0% of studies stating that taxonomic references were consulted to name ant species. At least 14.5% of ant diversity studies, or 29.0% that relied on the taxonomic literature for ant identification, alleged species were named following identification tools on taxonomic catalogues or online repositories. It is undoubtedly desirable that researchers in different knowledge areas search, retrieve and independently access the content of taxonomic publications to identify their study organisms. However, we detected some serious problems associated with this practice. There is a misinterpretation of the content of taxonomic catalogues, since these publications do not provide any kind of taxonomic tool for ant identification. Such works bring the taxonomic history of the taxa and, despite serving as reliable sources for taxonomic classification, cannot be used to identify species. So, two important questions arise. First, why do researchers confuse classification (i.e. the process of organizing categories following a logically structured system) with identification (i.e. the process of identifying properties of a given entity while ascribing it to a previously established category)? And the second is, if these authors did not identify their ants based on these catalogues, which are synoptic treatises of all known classificatory schemes in a given group, where do the names presented in their studies come from?

A similar limitation is detected in studies that present online repositories as primary or single identification sources. The leading online repository mentioned for ant identification is AntWeb.org [42]. In fact, AntWeb.org has revolutionized myrmecology since it was implemented online back in the 2000s, providing specimen-level data, images and natural history content linked by unique specimen identifiers directly to taxonomic names managed by the online catalogue of the ants of the world [24]. In other words, AntWeb has promoted, with unprecedented taxonomic accuracy, broad access of the scientific community to data otherwise restricted to old drawers of natural history museums and researchers' field books. Even so, inexperienced researchers’ use of the information available in AntWeb may lead to serious imprecisions in myrmecological literature, especially when they ignore the taxonomic literature and ‘identify’ their ant species based solely on the comparison of their specimens with photos available in the AntWeb platform. The image use in AntWeb to confirm or support ant identifications is highly recommended, especially when the users rely on the images of the type-specimens available therein. However, it should never be done as the first or only step toward species identification in any study.

Another concerning trend from our data is that authors cite outdated taxonomic publications despite more recent tools. For example, the use of ‘Bolton, 1994. *Identification guide to the ant genera of the world*’ [38]. Despite being a taxonomic landmark in the 1990s and 2000s, this work is consistently cited to the present day, although at a lesser frequency (figure 2*c*). Unfortunately, the classification proposed in this reference is basically irrelevant nowadays given recent updates in ant systematics. A possible reason for such tendencies may be that some authors cite taxonomic resources ‘by default’, probably based on previous publications when they relied on other identification methods in their studies. Despite the high number of citations of outdated catalogues and online repositories as primary identification sources, most of the taxonomic studies consulted by Brazilian myrmecologists contain proper identification tools. They consist of original taxa descriptions, taxonomic revisions and identification guides. Also, the high citation rates of comprehensive taxonomic studies (i.e. publications including identification keys to supraspecific categories) immediately after publication is evidence that they are readily absorbed by the myrmecological community (figure 2*c*). In this regard, it would be highly desirable for taxonomists to engage in more publications of comprehensive identification guides or updates to existing guides, including identification keys for higher taxonomic levels such as subfamilies and genera. Our data show that publications of this nature, accessible to researchers in Brazil, have been published at intervals of 9 to 12 years [19,33,38]. Considering the increasing frequency of reclassifications and publications describing new taxa, this time-lapse between the publication of identification guides certainly impacts the taxonomic resolution in different areas of knowledge.

Brazil can be considered a privileged country due to the high number of ant collections distributed, although unevenly, across the country. In our survey, 39.0% of studies indicated that their ants were identified by comparison with specimens deposited in at least 35 different ant collections. The two historically more traditional ant collections in Brazil, the MZSP and CPDC, are also the most sought after by Brazilian myrmecologists for the identification of their material. Nevertheless, several other traditional institutions and a few recent taxonomic centres have been increasingly considered for their potential as reference collections (figure 2*d*), expanding the possibilities for researchers to identify their material with the taxonomic precision expected from scientific works.

Besides keeping invaluable ant collections, the Brazilian institutions mentioned above have the mission to form a new generation of ant taxonomists. This is an extremely relevant role considering that most ant researchers working in the country are exclusively dedicated to community and behavioural ecology [29,30]. Thus, in addition to describing and organizing ant diversity, few taxonomists have historically been responsible for ant identification in Brazil. Here, we also show that 40.0% of studies published in the last 50 years on Brazilian ants stated that the species collected in their samples were identified with the support of taxonomists or experienced ant identifiers (figure 2*b*,*f*). The most important trend highlighted here is that 82 researchers from different knowledge areas were consulted to support ant identification in at least one study in the last 50 years. These findings suggest that the knowledge of ant identification in Brazil is not entirely restricted to a small number of overworked taxonomists. Since these non-taxonomist experts strive to provide accurate identifications with a solid taxonomic basis, the options for ecologists and ethologists seeking to apply names to their study objects can increase considerably. It is also important to highlight the increasing trend in the number of newly formed or emerging taxonomists identifying ants in Brazil (electronic supplementary material, table S4), especially in the current global context of taxonomy [43].

When evaluating the proportion of studies mentioning specimen vouchering, considering that taxonomic validation and scientific reproducibility are inseparable, we found that more than 60 institutions were listed as depository collections of the specimens captured in ant diversity studies in Brazil (figure 2*e*). A positive outcome of this study is the wide preference of researchers regarding depositing specimens in public access collections based in museums or universities. One of the 66 collections listed is private, where vouchers for two studies were deposited. There is also a division regarding the nature of the collections listed involving thematic laboratories and institutional collections. Altogether, 31 out of the 66 collections listed are restricted to research laboratories, maintained with resources from the coordinators, while 34 are institutional collections, presumably with broad access. This fact raises a relevant question regarding the access and longevity of these collections. In theory, museums and institutional collections in public universities tend to be longer-lived than collections restricted to thematic laboratories. This difference may raise concerns about the fate of these laboratory collections. Since researchers can move institutions, retire or even die, the future of these collections may be threatened in the absence of the human and financial resources necessary for their expansion and maintenance. Also, permission to access laboratory collections tends to fall to a single individual, the laboratory coordinator, while institutional collections tend to be more accessible or at least should be. In this sense, we can foresee an impact on the reproducibility of studies whose vouchers are deposited in collections with restricted access. Thus, it seems sensible to propose that researchers deposit the specimens of their studies in more than one collection away as possible, with a preference for broad-access collections.

Regarding the main repositories for ants in Brazil, from the 310 studies that explicitly indicated that the specimens collected were formally vouchered, only 5.8% included specimen accession codes in the main text or appendages. This low number represents a significant limitation in the reproducibility of these works, especially considering that, given the social nature and high abundance of ants, only a fraction of the individuals captured are entirely processed (i.e. sorted, mounted, labelled, identified, vouchered, databased and deposited) in myrmecological studies, with the remaining samples returning to their original vials or being simply discarded. As several ant genera are taxonomically challenging regarding morphological variation [44], the dissociation between processed individuals and replicates in the vials, represented by the lack of identifiers and accession codes, impairs the confirmation of ant identity and abundance in the studies. Authors should bear in mind that misidentifications are likely to occur with the dry-mounted specimens chosen to represent the identity of a given species in their studies and with the non-vouchered specimens in original samples. Vouchered mounted species may be a mixture of different species in the vials. An initial morphotyping of specimens into ambiguous entities in the study's beginning could also generate the same problem. Only one mounted specimen would represent a group potentially formed by multiple species. In the presence of these problems, studies would underestimate the local ant diversity by treating more than one species as the same entity within their sample. In addition, taxon concepts (as defined by [45]) are constantly being modified because of recent taxonomic evidence, and in these cases, reinterpretations of original data will be required [31]. Consequently, if the original samples cannot be tracked, this study's results can never be revised [13]. Another major concern is that several studies failed to present the location of depository collections. Although the leading institutions are well-known and widely accessible, some are organized in complex internal divisions (i.e. sections, unities, departments, campuses). Also, several minor and less traditional institutions cannot be accessed by readers based only on their names. To adopt an efficient vouchering policy, authors should provide the complete addresses and internal organization of their depository collections, preferentially including the name of the current curators or managers.

In addition to providing basic taxonomic information and specimen vouchering, authors should consider acknowledging the taxonomic background of their studies. Here we show that only 40.0% of ant diversity studies cited taxon authorities and years of description, at least in the first citation of species in the text (figure 2*a*). We recognize, however, that the absence of species' original descriptions year in diversity studies is not necessarily an author's failure, but may reflect the stylistic requirements of particular journals, which stipulate that only the authority's name should be provided at a taxon's first mention. On a related point, current metrics of scientific impact have been considered unfair regarding taxonomic research output [46]. This pattern has led some authors to suggest that references in which species were originally described should be cited in any study [47,48]. However, as pointed out by Meier [16], citing and including original species descriptions in the references may not be appropriate when these works are not adequate to permit identification (i.e. diminutive and incomplete species descriptions or inaccurate species delimitation) or when modern and accurate identification tools are available. Meier [16] argues that species identification should be formally treated as a method and reported as a result, and species descriptions cited only when including valid taxon concepts and identification tools. Zeppelini *et al*. [32] proposed a recent solution for this dilemma that suggests including full taxonomic references for the studies citing one or a few taxon authorities. For studies with many sampled species, of which the full reference of taxonomic works would result in a drastic increase in manuscripts length, an alternative would be to link the relevant taxonomic references either by a digital object identifier (DOI) or as discrete metafiles (in the form of supplementary material) that citation tracking databases would check. This inclusion would ensure that these references will be incorporated in impact metrics whenever cited. Specifically for ants, complete references for taxon authorities can be readily retrieved from Bolton's online catalogue [24]. Whatever solution an author chooses, the intellectual contribution of previous taxonomic works should be cited to ensure appropriate recognition of taxonomy as a fundamental biological discipline.

Yet, another concerning tendency from our survey is that identification guides to ant subfamilies and genera have been cited as a proxy for species identification in most studies. In these cases, authors of studies that bring species lists simply state that ants were identified using the keys in the work ‘X’ or ‘Y’ (here mostly the references [19,33,38]) even when these keys do not allow for the identification of ants at a specific level, and no other taxonomic work is mentioned. When authors mention identification at the species level, they usually state that ants were identified using the ‘relevant literature’ or the ‘taxonomic literature’, but no taxonomic work containing keys to species is cited or referenced. The problems with this approach are obvious. By not citing the taxonomic works used to identify the species, authors not only neglect basic taxonomic information that should allow reproducibility, but also intensify the distance between taxonomists and other researchers regarding citation and impact factor metrics [16].

Along with adequately referencing the taxonomic works and mentioning taxonomists and collections consulted to identify specimens, authors from different knowledge areas can embrace taxonomy by joining taxonomists' efforts to publish their studies. In Brazil, this practice has been increasingly incorporated into the routine of ant researchers. In the last 50 years, we observed that around 30.0% of the ant diversity studies on Brazil had taxonomists among their authors. Despite the broad international network evidenced by our results, ant researchers in Brazil tend to seek national institutions for collaboration (figure 3). That is an expected outcome considering that researchers will select local cooperators because of their regions’ natural affinities and ant fauna similarities. We also show that the Brazilian institutions that house the main myrmecological collections concentrate the highest values of inter-institutional collaboration (figures 2*d,e* and 3). Part of these collections is associated with the historical presence of taxonomists, who are frequently sought after for species identification [49–51]. In sum, traditional collections usually harbour taxonomists and a high diversity of taxa deposited, facilitating species identification by comparison, and promoting inter-institutional connections. However, establishing and expanding myrmecological collections in new institutions should be permanently encouraged.

Considering the temporal patterns observed here, Brazil's proportion of ant diversity studies explicitly providing basic taxonomic information has slightly increased in the last few years. However, the frequency of mentions in the literature varies among the distinct methods authors apply to identify their ants (figure 2*b*). The frequency of studies that relied on the taxonomic literature, reference collections and specialists for ant identification has marginally increased in the last decades, although with many variations over time (figure 2*b*,*d*,*f*). Similarly, despite only six collections concentrating most of the specimen vouchering in Brazilian ant diversity studies, there was an increase in the number of institutions available for voucher depositing in Brazil in the last two decades (figure 2*e*; electronic supplementary material, table S5). Still, the frequency of specimen vouchering in Brazilian ant collections has visibly changed recently, with a gradual reduction of voucher depositing in the more traditional institutions (CPDC, MZSP and INPA) in the last 10 years (figure 2*e*, red, blue and orange lines), and an increase in voucher depositing in the newly established DZUP collection (figure 2*e*, green line) at the same period. This apparent reversal highlights both the importance of establishing new institutions with the mission of adequately housing biological collections and the need to invest in the maintenance and expansion of traditional collections through the input of financial resources and the hiring of taxonomists.

Although somewhat discrete, the positive tendencies found here can undoubtedly result from the recent appeals in biological literature toward more transparent scientific practices [9,10,16,31]. These scientific advances are presumably an effect of the increased availability of resources for identifying ants in recent decades [19,33,38], in addition to the consequences of a period of high investment by the Brazilian government in the expansion of educational and research institutions between 2004 and 2016 [55]. Also, the increasing collaboration between ant researchers from different knowledge areas and taxonomists [14,30] contributed to these advances in Brazil. However, the collaboration between areas is way beyond the simple recognition of taxonomic work [52]. Considering all the information presented here regarding the taxonomic validation of diversity and conservation studies, it is natural that the united effort of ecologists and taxonomists has the potential to improve the scientific quality of both disciplines through a most accurate insight into the organisms and improved feasibility of data sampling at large scales and on more diverse and functionally important groups [53]. It also improves the prospects of all scientists involved, opening potential funding sources and helping to solve the so-called ‘taxonomic impediment’, as suggested by the data of our study. Also, we cannot ignore that different informal estimates indicate that the number of ant species in the world could reach 20 000 [54], at least 6000 more species than the richness currently known [24]. Part of the ant diversity that remains to be described probably has already been collected in ecological inventories. A closer relationship between ecologists and taxonomists can decrease this taxonomic gap by making the undescribed diversity available for comprehensive taxonomic studies, which in turn have the potential to update identification tools.

Our results are relevant not only within the scope of Brazilian myrmecology. Biological organisms are study models in the most distinct fields, and the lack of information guaranteeing strict identification procedures is a usual failure in biological sciences [17]. Currently, large datasets in ecology, composed of hundreds or even thousands of taxa, are increasingly being assembled in the most diverse biology areas [56,57]. Nevertheless, the value of such datasets for addressing research questions is first determined by the taxonomic accuracy underpinning their taxa names [13]. Also, sampling, processing, identifying and depositing biological specimens is not exclusive to ant diversity studies. Ants are well-known social insects and, therefore, often collected in relative abundance. Thus, it is logical to conclude that the patterns emerging from our results are even more significant for diversity studies on solitary organisms or taxa collected in low abundances, since the availability of replicates for future access is considerably limited in these cases.

## 5. Conclusion

The survey and critical analysis of the taxonomic information in ant diversity studies published in the last 50 years in Brazil highlight that this scientific community tends to accomplish the minimum taxonomic criteria for scientific reproducibility in most cases. The proportion of studies that provide taxonomic data in sufficient detail to permit precise validation of taxonomic identifications is relatively high compared with the global trends for non-taxonomic studies. These positive aspects have evolved to become more common among the new generations of myrmecologists in Brazil. However, there is a lot of room for improvement. More attention must be given to the appropriate vouchering of species in repositories, preferably with accession numbers allowing ready detection for future confirmation. Additionally, with less than half of the studies providing taxon authorities and years of description and the evident lack of recognition of taxonomic works containing identification keys to species, an urgent change is needed. Ant researchers in Brazil must give due credit to taxonomy, the science responsible for supporting all work in the most different fields of biology.

In conclusion, we follow previous studies [10,15,17] in advocating that researchers and journals must meet minimum criteria to publish in any area of knowledge involving organisms. In our case, we advocate for four minimum standards: (i) providing the complete methods used for the identification of all studied taxa; (ii) compromise in indicating and explicitly referencing each taxonomic work that underpinned studies main conclusions; (iii) presenting the name, address and contact of all specialists and collections consulted for taxonomic identification if they are not authors of the work; and (iv) deposit of voucher specimens in open-access collections and provide accession numbers that can be queried for future investigations. Finally, but not less critical, authors must bear in mind that collaborations between ecologists and taxonomists can substantially increase the taxonomic resolution of ecological papers, improving the quality and effectiveness of nature conservation and management plans.

## Data Availability

Compiled data were arranged in the electronic supplementary material [58], including the list of studies retrieved from online repositories and values of taxonomic descriptors extracted from each study. Data used to generate statistics and figures, including supporting metadata with descriptions and explanations of the abbreviations used in each dataset are available from the Figshare digital repository: https://doi.org/10.6084/m9.figshare.18865298.
